# *Pectobacterium parmentieri* SCC 3193 Mutants with Altered Synthesis of Cell Surface Polysaccharides Are Resistant to N4-Like Lytic Bacteriophage ϕA38 (vB_Ppp_A38) but Express Decreased Virulence in Potato (*Solanum tuberosum* L.) Plants

**DOI:** 10.3390/ijms22147346

**Published:** 2021-07-08

**Authors:** Przemyslaw Bartnik, Sylwia Jafra, Magdalena Narajczyk, Paulina Czaplewska, Robert Czajkowski

**Affiliations:** 1 Laboratory of Biologically Active Compounds, Intercollegiate Faculty of Biotechnology UG and MUG, University of Gdansk, Antoniego Abrahama 58, 80-307 Gdansk, Poland; bartnikprzemyslaw@gumed.edu.pl; 2 Laboratory of Plant Microbiology, Intercollegiate Faculty of Biotechnology UG and MUG, University of Gdansk, Antoniego Abrahama 58, 80-307 Gdansk, Poland; sylwia.jafra@ug.edu.pl; 3 Laboratory of Electron Microscopy, Faculty of Biology, University of Gdansk, Wita Stwosza 59, 80-308 Gdansk, Poland; magdalena.narajczyk@ug.edu.pl; 4 Laboratory of Mass Spectrometry-Core Facility Laboratories, Intercollegiate Faculty of Biotechnology UG and MUG, University of Gdansk, Antoniego Abrahama 58, 80-307 Gdansk, Poland; paulina.czaplewska@ug.edu.pl

**Keywords:** phage receptor, LPS, soft rot, endotoxin, pectinolytic *Erwinia*, *Pectobacterium wasabiae*, *Pectobacterium parmentieri*

## Abstract

*Pectobacterium parmentieri* is a Gram-negative plant-pathogenic bacterium able to infect potato (*Solanum tuberosum* L.). Little is known about lytic bacteriophages infecting *P. parmentieri* and how phage-resistance influences the environmental fitness and virulence of this species. A lytic phage vB_Ppp_A38 (ϕA38) has been previously isolated and characterized as a potential biological control agent for the management of *P. parmentieri*. In this study, seven *P. parmentieri* SCC 3193 Tn5 mutants were identified that exhibited resistance to infection caused by vB_Ppp_A38 (ϕA38). The genes disrupted in these seven mutants encoded proteins involved in the assembly of O-antigen, sugar metabolism, and the production of bacterial capsule exopolysaccharides. The potential of A38-resistant *P. parmentieri* mutants for plant colonization and pathogenicity as well as other phenotypes expected to contribute to the ecological fitness of *P. parmentieri*, including growth rate, use of carbon and nitrogen sources, production of pectinolytic enzymes, proteases, cellulases, and siderophores, swimming and swarming motility, presence of capsule and flagella as well as the ability to form biofilm were assessed. Compared to the wild-type *P. parmentieri* strain, all phage-resistant mutants exhibited a reduced ability to colonize and to cause symptoms in growing potato (*S. tuberosum* L.) plants. The implications of bacteriophage resistance on the ecological fitness of *P. parmentieri* are discussed.

## 1. Introduction

The Soft Rot *Pectobacteriaceae* (SRP): *Pectobacterium* spp. and *Dickeya* spp. (former pectinolytic *Erwinia* spp.) are broad-host range agricultural pathogens causing disease symptoms in staple food crops worldwide [1,2]. SRP bacteria cause significant losses in crop production (up to 40%), with disease severity dependent on weather conditions, plant susceptibility, and the abundance of pathogen inoculum [3]. In potato (*Solanum tuberosum* L.), SRP bacteria are responsible for tuber soft rot in transit and storage and potato blackleg in field-grown plants [1]. Among the economically significant hosts are potato, carrot, tomato, onion, pineapple, maize, rice, hyacinth, chrysanthemum, and calla lily [4]. SRP bacteria are recognized as among the top ten most important pathogens of plants [5].

Together with other SRP species causing diseases of crops, *P. parmentieri* (formerly known as *Erwinia carotovora* subsp. *wasabiae* and later *P. wasabiae*) has recently been recognized as an increasing threat for potato cultivation [6,7,8]. This species was first described as a pathogen in 2010, causing severe potato blackleg outbreaks in field-grown crops in New Zealand [9]. In the following years, the pathogen was frequently reported both in symptomatic and symptomless potato plants cultivated in many countries, both in warm as well as cold climates, including Canada, Iran, South Africa, Zimbabwe, and most European countries [10]. Due to the development of new genome-based taxonomic classification techniques, it has become evident that many bacteria previously characterized as *Pectobacterium carotovorum* that were isolated from crops in Europe and elsewhere should be currently re-classified as *P. parmentieri* [11]. In the last ten years, the dominant role of *P. parmentieri* in the epidemiology of potato blackleg and soft rot diseases in Europe and worldwide has been widely recognized [8].

Management of potato diseases caused by SRP, including *P. parmentieri*, is complicated and largely ineffective [12]. Most commercial potato cultivars are susceptible to blackleg and soft rot caused by SRP bacteria, and immune (resistant) potato cultivars have not been developed [13]. The pathogen may quickly spread both within an agricultural field and between fields since it often initially causes only very mild and/or latent infections in growing plants and progeny tubers [2]. Pathogen detection is also hampered by a lack of specific and sensitive enough detection tools and protocols [12].

Lytic bacteriophages (synonym: phages, bacterial viruses) can be considered to be an environmentally friendly alternative to standard (chemical and physical) plant protection strategies used in agriculture to prevent crop spoilage [14]. Likewise, the bacterial viruses of *Pectobacterium* spp. and *Dickeya* spp. have been already evaluated in several studies targeting isolation and characterization of new viruses, biological control of their host bacteria in agricultural applications and molecular host–phage interactions in the environment [14,15,16,17,18,19,20,21,22].

We previously isolated the lytic bacteriophage vB_Ppp_A38 (ϕA38) that infected only *P. parmentieri* and described its features as necessary when applying it as a biological control agent in potato. Phage vB_Ppp_A38 (ϕA38) is a member of the N4-like family *Schitoviridae*, genus *Cbunavirus*. Genetically ϕA38 is the most similar to the bacteriophages vB_PatP_CB1, vB_PatP_CB3 and vB_PatP_CB4 infecting *P. atrosepticum* [23].

In the proof-of-concept experiments, this bacteriophage protected potato tubers inoculated with *P. parmentieri* from rotting [24,25].

To effectively use bacteriophages for the biological control of disease and to better assess the ecological role of bacterial viruses both in natural and agricultural environments, detailed knowledge of the molecular basis of phage interactions with their bacterial hosts is necessary. As all bacteriophages are obligate parasites of bacteria, they are totally dependent on their bacterial hosts to reproduce and thrive in a given ecosystem [26]. Consequently, the persistent propagation and ecological success of bacterial viruses are strictly dependent on their capability to infect bacterial hosts and, once infection occurred, to complete their life cycles, producing progeny (daughter) viruses. Likewise, as a consequence of their success in infecting hosts, lytic bacteriophages can strongly influence the abundance and diversity of susceptible bacteria in the environment [27]. Thus, these viruses exert selective pressure on their hosts, promoting the spread of strains with reduced fitness [28,29]. This reduced fitness is often due to the modification or loss of structures present on the surface of host cells that can be important for virulence, such as receptors necessary for infection. Therefore, bacterial strains resistant to phage infection are often also less virulent and/or less fit [30,31].

Although many studies have focused on analyses of the phage attachment process and bacterial resistance against viral infections on a molecular level, most such investigations have only addressed interactions of a few model bacterial viruses (e.g., phages λ and T4) and model bacteria such as *Escherichia coli* [32]. The knowledge of these processes in plant-associated and plant-pathogenic bacteria, including SRP and their bacteriophages, is still largely missing [33]. It is unknown, for example, whether phage-resistant SRP bacterial strains are more or less fit [34] in their natural environment, potato plants. Thus, the presence of phages might lead to indirect benefits to crop production by selecting for less virulent strains (variants) of pathogens. Although the emergence of phage-resistant bacterial variants under natural conditions is probably inevitable [35], research linking phage-resistance and host ecological fitness, especially in SRP, has not been performed. The purposes of this study were: (i) to identify and characterize those *P. parmentieri* genes encoding bacterial structures required for vB_Ppp_A38 (ϕA38) attachment and susceptibility to infection and (ii) to evaluate whether disruption of such genes in *P. parmentieri* also result in altered phenotypes of the bacterial mutants, primarily decreased ecological fitness, and reduced ability to cause disease symptoms *in planta*.

## 2. Results

### 2.1. Transposon Mutagenesis and Identification of Tn5 Disrupted Genes in ϕA38-Resistant P. parmentieri SCC 3193

The 1000 mutants were randomly selected from the mutant pool and screened for resistance to infection caused by bacteriophage vB_Ppp_A38 (ϕA38). A total of seven Tn5 mutants were found to be resistant to ϕA38. The genomes of phage-resistant bacterial mutants were sequenced to identify the transposon insertion sites and their accompanying genomic neighborhood (Table S1, Supplementary Materials). A single Tn5 transposon insertion site was observed in each of the 7 mutants.

### 2.2. Characterization of the Disrupted Genes, Transcriptional Organization, Biochemical Pathways and Cellular Enzymatic Networks Conferred by Tn5 Insertion

The loci disrupted by Tn5 in ϕA38-resistant SCC 3193 mutants encoded proteins involved in (i) assembly of O-antigen (mutants M26, M205, M392, M603, M649), (ii) metabolism of sugars (mutant M465), and (iii) production of bacterial capsule exopolysaccharide (EPS) (mutant M4) (Table 1). The sequences bordering the inserted Tn5 transposons of 7 mutants (targeting ca. 1000–5000 bp.) were analyzed using BlastP to acquire additional insights into their genomic context. Of these seven regions that were interrogated for their transcriptional organization, 2 insertions (*p4* and *p26*) were expected to be transcribed as individual genes, whereas 5 other insertions (*p205*, *p392*, *p465*, *p603*, and *p649*) were predicted to be parts of operons. Examination of KEGG biochemical pathways corresponding to these 7 transcriptional units enabled their assignments to cellular pathways involved in cell wall and capsule biogenesis and biosynthesis of cell surface lipopolysaccharides and exopolysaccharides (Table 1).

### 2.3. Adsorption of ϕA38 to Viable and Non-Viable Host Cells

**Figure 1:**
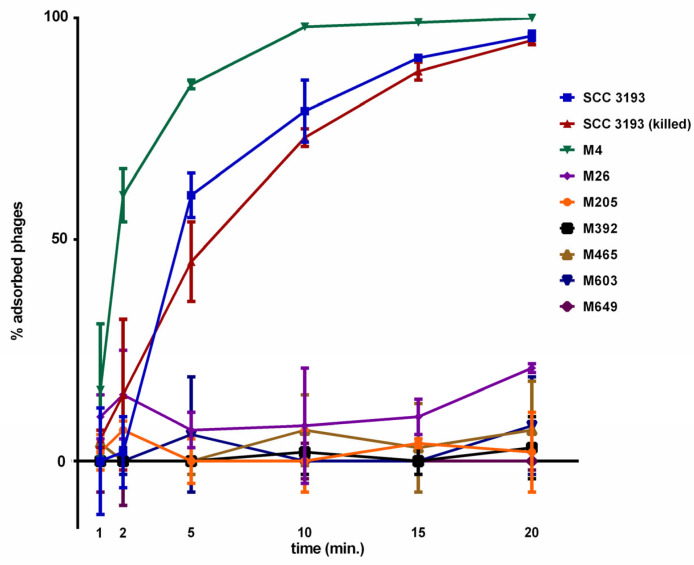
Adsorption of ϕA38 to viable WT *P. parmentieri*, WT killed with chloramphenicol, and seven phage-resistant mutants. A MOI of 0.01 of ϕA38 was used for adsorption assay and the total assay time was 20 min. Phage adsorption was calculated as follows: the percentage adsorption = (the average titer of unabsorbed phages per sample/average titer of phages in negative control) × 100. The averages together with standard deviations of three independent repetitions per strain (WT or mutants) are shown.

The adsorption of vB_Ppp_A38 (ϕA38) to wild-type (WT) cells of *P. parmentieri* SCC 3193 was fast (Figure 1). Within the first 5 min ca. 60% of phage particles has adsorbed to the host cells, and after 20 min, more than 96% of ϕA38 particles had adsorbed to host SCC 3193 wild-type cells.

The rate of adsorption of ϕA38 to cells of this WT strain that were killed by antibiotic was similar to that of viable WT cells (Figure 1). Furthermore, the rate of adsorption of ϕA38 to mutant M4 was similar to that of the WT strain (Figure 1). In contrast, the adsorption of ϕA38 to the other 6 mutants was almost completely abolished. In these mutants, only 2 to 10% of the phage particles had adsorbed by 20 min, with the rest remaining free (Figure 1).

**Figure 2:**
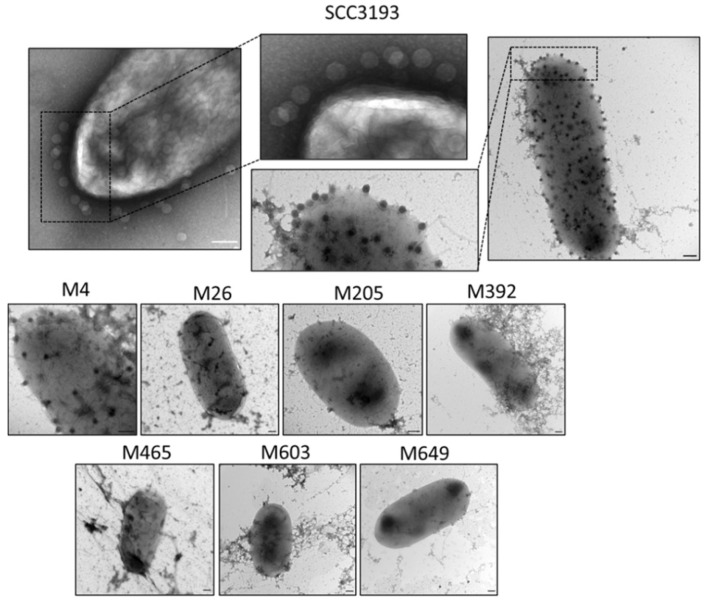
Visualization of adsorption of ϕA38 phage particles to wild-type *P. parmentieri* and phage-resistant mutants by transmission electron microscopy (TEM). Bacterial cells and phage particles were mixed at MOI of 10 and incubated for 20 min at 20–22 °C to allow the phages to attach to host cells. At least 10 individual images were collected for each strain, and the experiment was repeated once (two biological replicates of the assay). Representative photos are shown. Scale bar—200 nm.

Transmission electron microscopy was also used to visually assess the interaction of ϕA38 with wild-type and phage-resistant mutants. The abundant adsorption of ϕA38 to the surface of both WT cells and mutant M4 cells was observed. In contrast, no adsorption of phage particles to any of the other 6 phage-resistant mutants was detected (Figure 2).

### 2.4. Identification and Characterization of the Crude Lipopolysaccharide (LPS) Isolated from P. parmentieri Strain SCC 3193 Wild-Type and Tn5 Mutants

**Figure 3:**
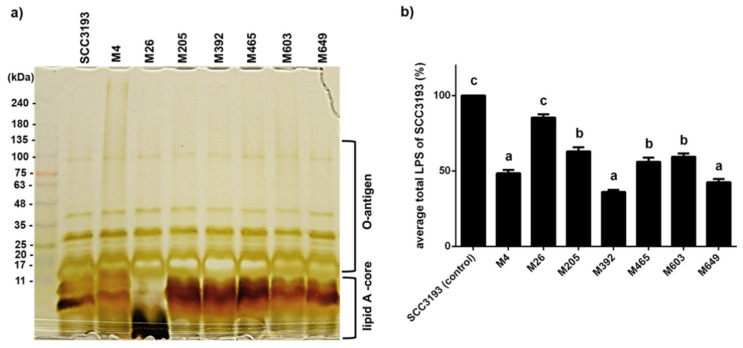
Characterization of lipopolysaccharide (LPS) from wild-type *P. parmentieri* strain SCC 3193 and seven phage-resistant mutants. (**a**) SDS-PAGE was performed using a gradient (4–20%) polyacrylamide gel, and the LPS components were visualized by silver staining [37]. The size marker (11–245 kDa, Perfect Tricolor Protein Ladder, EURx, Poland) is shown in the first lane. (**b**) Quantification of the total LPS produced by wild-type SCC 3193 strain and seven phage-resistant bacterial mutants was done using Pierce™ LAL Chromogenic Endotoxin Quantitation Kit (ThermoFisher Scientific, Warsaw, Poland) according to a protocol provided by the manufacturer. The concentration of LPS of the Tn5 mutants is expressed as % of the LPS amount produced by the wild-type SCC 3193 strain. The means that do not share the same letters above each bar differ (*p* = 0.05). The experiment was repeated three times (each biological replicate contained two technical replicates), and the results were averaged for analysis. Vertical lines represent standard deviation (SD).

Given that mutations in several of the phage-resistant mutants suggested that surface polysaccharide production might be altered, preparations of crude LPS from WT strain SCC 3193 and the 7 phage-resistant mutants were compared using SDS-PAGE. The LPSs from all tested strains were resolved into many bands of various mobilities (Figure 3a). The putative lipid A-core of the LPS derived from the WT strain was composed of two heavy bands, both with masses less than 11 kDa, one faint band with a mass between 11 and 17 kDa, and three faint bands with masses between 17 and 25 kDa. The putative O-antigen component of the SCC 3193 WT LPS was composed of 8 (faint) bands with masses between 25 and 100 kDa. All LPSs purified from the phage-resistant mutants, except that from M26, resembled that of the wild-type strain. The LPS of mutant M26 lacked the two bands with masses less than 11 kDa constituting the putative lipid A-core but possessed a very small mass band of ca. 2–3 kDa. The putative O-antigen component of the LPS of mutant M26 was similar to that of the wild-type strain (Figure 3a, Figure S3). A significantly lower amount of LPS was detected in the other 6 phage-resistant mutants compared with that in the WT strain. The most significant reduction in total LPS was observed in mutants M392 and M649, which produced only ca. 37% and 44%, respectively, of the LPS produced by the SCC 3193 WT strain. Mutants M4, M205, M465, and M603 produced about 50% to 65% of the LPS produced by the WT strain. Although the composition of the LPS of mutant M26 was apparently altered compared to the WT strain, its content of LPS was reduced by only about 13% compared with the WT strain (Figure 3b).

### 2.5. Mass Spectrometry Analysis of the P. parmentieri SCC 3193 and Tn5 Mutants’ Surface Proteins

**Figure 4:**
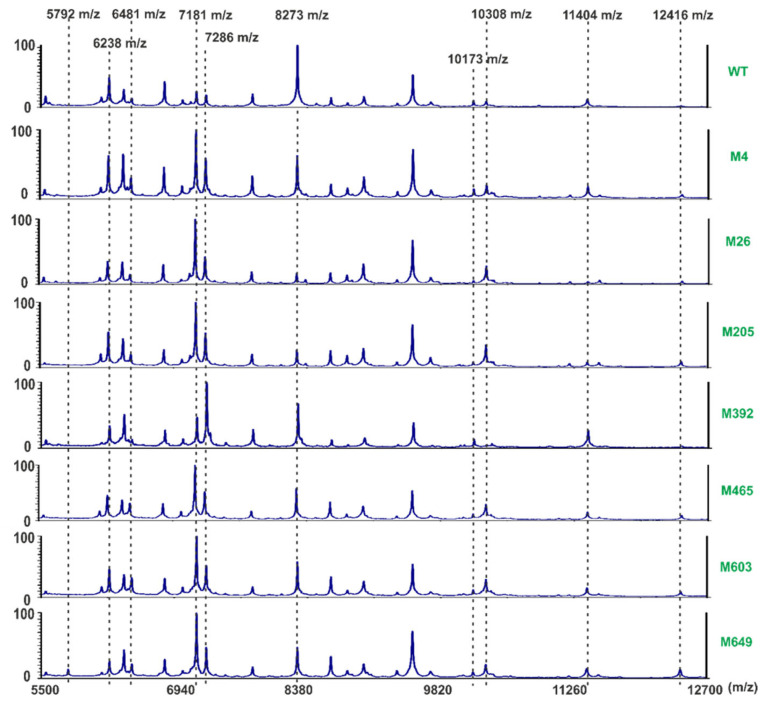
Representative MALDI-TOF spectra of *P. parmentieri* wild-type strain SCC 3193 and seven phage-resistant mutants obtained with a sinapinic acid (SA) matrix. Averages from two independent biological replicates, each containing three technical replicates, are shown.

Whole-cell MALDI-TOF fingerprinting technique was used to compare the bacterial surface proteins of the WT strain with these of the phage-resistant mutants. The initial optimization of the ranges of MS recorded masses revealed that the differences in the recorded m/z signals were the most discriminative in the ranges between 5000 and m/z. Initially, two matrices (DHB-2,5-dihydroxybenzoic acid and SA-sinapinic acid) were compared. Optimum separation was obtained using the sinapinic acid matrix, and all analyses used this MS matrix. MS measurements were repeated three times for each strain. The experiment was independently repeated one time with the same setup. The averaged measurements (m/z signals) of each strain were determined. Inspection of the obtained spectra revealed a high similarity between all bacterial strains tested (Figure 4). More detailed analyses revealed specific signals that discriminated against the strains. Mutant M649 was distinctive in exhibiting a signal of 5792 m/z. Phage-resistant mutants differed in the presence and intensity of signals with m/z values of 6238, 6396, and 6481 m/z. The SCC 3193 WT strain harbored relatively low amounts of proteins with m/z values of 7181 and 7286 compared to all Tn5 mutants resistant to infection caused by ϕA38.

Furthermore, in mutant M392, the protein with a 7286 m/z was more abundant than that having a mass of 7181 m/z. Likewise, the abundance of the protein with a mass of 8273 m/z was higher in the WT than in all mutant strains. Mutants M26, M205, M465, M603, and M649 each forward a higher abundance of a protein with a mass of 10,308 m/z than the WT strain, although this protein was in very low abundance in mutant M392. This mutant, however, contained a particularly large amount of protein with a mass of 11,404 m/z compared to other strains. Although the proteins with a mass of 12,416 m/z was present in all strains, they were in the highest abundance in mutants M603 and M649.

### 2.6. Inactivation of ϕA38 by LPS

To examine whether vB_Ppp_A38 (ϕA38) can directly use LPS as a sole receptor for adsorption to the *P. parmentieri* cells, phage inactivation in the presence of LPS was examined using LPS purified from the SCC 3193 WT strain. ϕA38 particles were not inactivated by the purified LPS.

### 2.7. Phenotypes of P. parmentieri Phage-Resistant Mutants

The seven Tn5 mutants resistant to infection caused by ϕA38 phage were tested for various phenotypes that might differ from that of the wild-type strain. No differences were observed among mutants and WT strain in most metabolic phenotypes examined using BIOLOG GENIII and EcoPlate phenotypic microarrays. The mutants differed from the wild-type strain in a total of only 5 features out of 94 tested with BIOLOG GEN III plates and from 31 tested using BIOLOG EcoPlates. Mutant M26 lost its ability to use both α-D-lactose and D-galactose as a sole carbon source and become susceptible to sodium butyrate. Mutant M205 lost its ability to use glucuronamide. Mutants M205, M392, and M465 become sensitive to 4% NaCl, and mutant M603 became susceptible to acetic acid. Although the WT strain was able to develop cavities on CVP medium, produced proteases, and was able to degrade carboxymethylcellulose and polygalacturonic acid, it was unable to produce siderophores and to grow on TSA medium supplemented with 5% NaCl, all but one Tn5 mutant maintained these phenotypes; mutant M26 failed to use polygalacturonic acid and did not produce proteases. Moreover, mutant M26 expressed a significantly increased ability to form biofilm compared to the WT strain. All other phage-resistant mutants retained the ability to form biofilm in a similar abundance as the WT strain. No differences in cell morphology were noted in the phage-resistant mutants compared to the *P. parmentieri* strain SCC 3193 using transmission electron microscopy.

**Figure 5:**
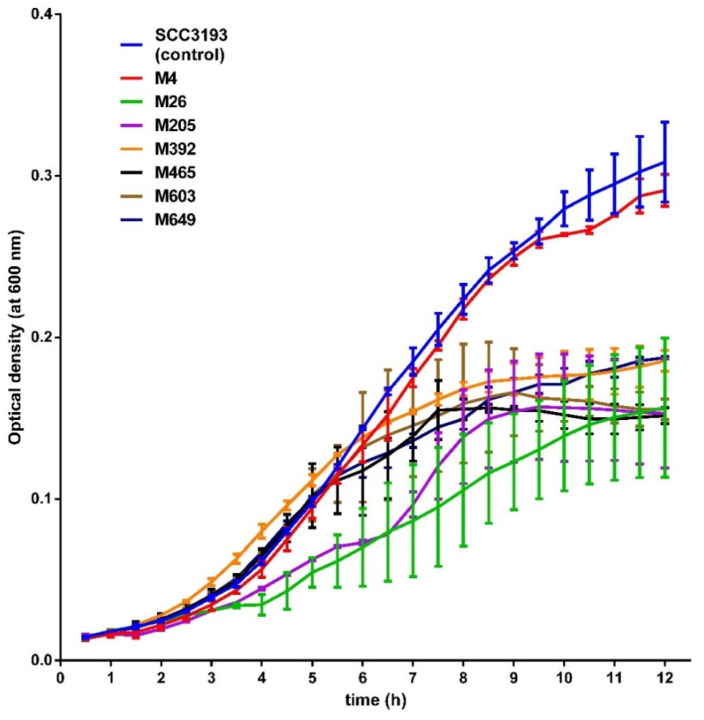
Growth of wild-type *P. parmentieri* SCC 3193 and phage-resistant mutants at pH 5.0. The experiment was performed in two biological replicates each containing two technical replications (*n* = 4). The results were averaged for presentation, the bars show standard deviation (SD).

Additionally, all mutants exhibited a similar colony morphology and diameter to that of the WT strain. None of the tested mutants differed significantly in their average generation times in either rich or minimal media. Likewise, the analysis of mutants’ growth at six different temperatures (5, 8, 15, 28, 37, and 42 °C) did not reveal any differences in growth compared to that of the WT strain, i.e., all tested strains were able to grow at 8, 15 and 28 °C but were unable to grow at 5, 37 and 42 °C. All phage-resistant mutants, except mutant M4, exhibited statistically significantly delayed growth at pH 5.0 compared to the WT strain (Figure 5). The growth of the mutants at pH 10.0 did not differ from that of the WT strain (data not shown). Likewise the WT strain, all phage-resistant mutants exhibited no swarming motility. Mutants M26, M205, M392, and M465 did not display swimming motility, unlike the *P. parmentieri* SCC 3193 strain (Figure S1). Although flagella were detected in the WT strain SCC 3193 and mutants M205, M465, M603, and M649, mutants M4, M26, and M392 lacked flagella (Figure S2). The WT strain and all phage-resistant mutants had a similar macroscopic capsule structure. All mutants expressed similar susceptibility/resistance to antibiotics as the SCC 3193 WT strain.

### 2.8. Virulence of P. parmentieri Tn5 Mutants

**Figure 6:**
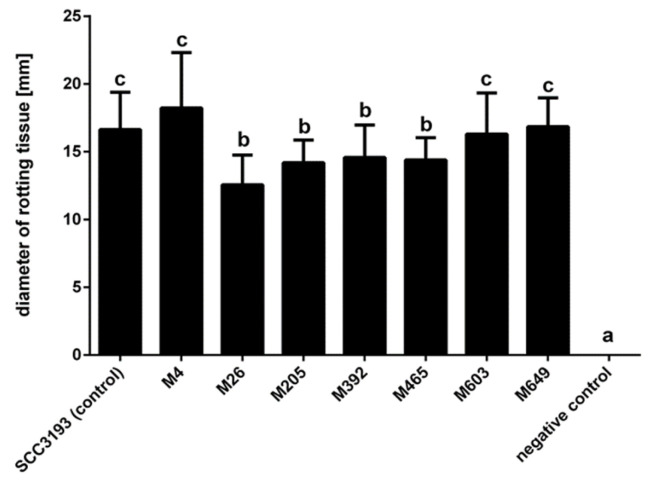
Maceration of potato tuber tissue by phage-resistant *P. parmentieri* mutants. The average diameter of rotting (in mm) measured after 72 h incubation at 28 °C in a humid box is shown. Three individual potato tuber slices were inoculated per mutant in two independent experiments (*n* = 18; three wells per strain per slice and per experiment). Results considered significant at *p* = 0.05 and pair-wise differences were obtained using the *t*-test. The means that do not share the same letters above each bar differ. Vertical lines represent standard deviation (SD).

Although all phage-resistant mutants exhibited some ability to macerate potato tuber tissue, mutants M26, M205, M392, and M465 were less virulent than the *P. parmentieri* SCC 3193 WT strain (Figure 6).

The ability of the mutants and WT strain to cause blackleg symptoms in potato plants after soil infestation was also investigated. In 2 separate phytochamber experiments with plants grown in potting compost, most potato plants inoculated with Tn5 mutants did not show any disease symptoms.

In contrast, between 20 and 40% of the plants inoculated with the WT strain developed severe and typical blackleg symptoms leading to the death of the inoculated plants. No symptoms were observed at any time of both experiments in plants inoculated with sterile Ringer’s buffer (negative control).

**Figure 7:**
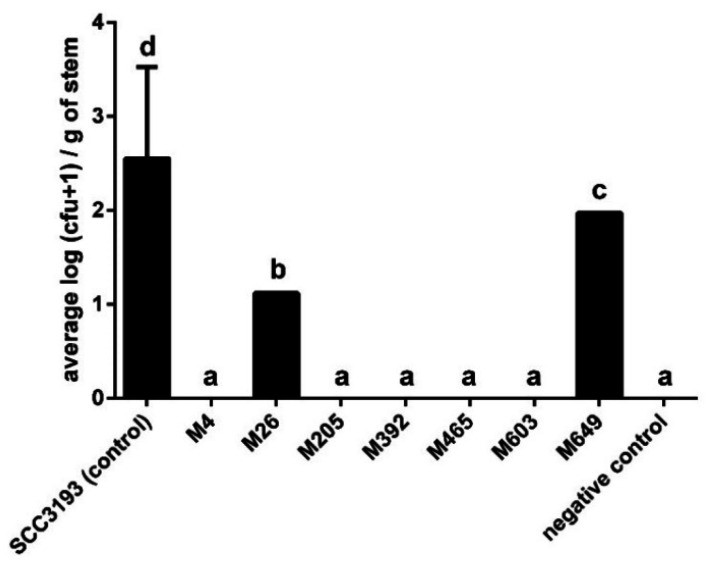
Population size of WT *P. parmentieri* strain SCC 3197 and seven phage-resistant mutants within stems of potato plants after inoculation into soil. Results considered significant at *p* = 0.05, and the pair-wise differences were obtained using the *t*-test. The means that do not share the same letters above each bar differ. Vertical lines represent standard deviation (SD).

The population size of the wild-type SCC 3193 pathogen differed significantly between plants, but the bacterial cells were recovered from 80 to 100% of the plants at 14 days after inoculation in 2 experiments at densities that ranged from 8–2000 CFU g^−1^ of stem tissue (Figure 7). As expected, stems of negative control plants did not harbor *P. parmentieri*. A much lower frequency of recovery of phage-resistant mutants from stems after soil inoculation was observed in both experiments. Mutant strains were detected in only two plants expressing typical blackleg symptoms; one plant harbored M26 mutant (130 CFU g^−1^ of stem tissue), and one plant harbored mutant M649 (ca. 900 CFU g^−1^ of stem tissue). None of the other mutants was detected inside potato stems.

## 3. Discussion

Although several studies have already addressed phage-resistance in the context of ecological fitness of bacterial hosts [38,39], the molecular mechanisms governing the interaction of plant-pathogenic bacteria and especially SRP with their (lytic) bacterial viruses and its ecological relevance remain relatively poorly understood [21,40]. For example, only limited information is available concerning the molecular basis of the phage-host binding process and the receptors used by (lytic) bacteriophages to infect *Pectobacterium* spp. and *Dickeya* spp. hosts [18,41]. In this study, we used a random transposon mutagenesis approach to identify those *P. parmentieri* genes that encode structures that are used by lytic bacteriophage vB_Ppp_A38 (ϕA38) for infection. Furthermore, the collection of the ϕA38-resistant *P. parmentieri* Tn5 mutants was assessed for various phenotypes likely to be involved in the ecological success of the pathogen, including its ability to cause disease symptoms in planta. Using this approach, we investigated the hypothesis that ϕA38-resistant *P. parmentieri* Tn5 mutants may be at a fitness disadvantage to the wild-type strain, and the level of this trade-off (host–phage resistance vs. host ecological fitness) is dependent on the environmental context [35].

All seven phage-resistant mutants found in our study had disruptions in transcriptional units (genes/operons) that were previously linked to phage adsorption in different species of Gram-negative bacteria [42]. The transposon insertions were in transcriptional units encoding enzymes involved in the synthesis and/or maturation of cell surface polysaccharides and the metabolism of cell surface sugar derivatives, including lipopolysaccharides and exopolysaccharides (Table 1). As no mutants have transposon insertions in other genes, unrelated to the synthesis of such cell surface features were found in our study, we assume that such cell surface components are a prerequisite for infection.

Knowledge of the role of bacterial surface polysaccharides, including LPS and EPS, comes primarily from studies done on human and animal pathogens. In contrast, the function of bacterial surface polysaccharides in plant-pathogen interactions is less understood [43]. In the mutant M4, the gene encoding putative O-antigen LPS acetylase (putative *oafA*/*yrhL*) involved in polysaccharide synthesis was disrupted. Although the function of this gene in *P. parmentieri* is not clear, the homologs of *oafA* in human pathogens, *Staphylococcus aureus* and *Salmonella typhimurium* codes for stress-related proteins which binds to cell surface polysaccharides and provide protection against host immune system during infection [44,45]. The *galU* gene encoding UTP-glucose-1-phosphate uridylyltransferase, which is disrupted in mutant M26, was reported to be required for the synthesis of capsular polysaccharides in *Pseudomonas syringae*. The *P. syringae* *galU* deficient mutant was affected in the development of disease symptoms in plants, survival in harsh environments, and was impaired in swimming motility [46]. In enteric bacteria and *Dickeya* spp., mutations in *galU* were linked with reduced fitness under stress conditions, a nonmotile phenotype, reduced secretion of flagellin and decreased ability to secrete proteases [47,48]. Most of these phenotypes were confirmed here in mutant M26. The *pseH* gene was disrupted in mutant M205. PseH is involved in the glycosylation of flagellin in *Campylobacter jejuni*, and a *pseH* deficient mutant lacked flagella and was nonmotile [49]. The ecological role of the PseH in *P. parmentieri* and other soft rot *Pectobacteriaceae* species remains unknown. It is noteworthy, however, that mutant M205 was unable to colonize and cause symptoms in *S. tuberosum* plants, suggesting that PseH plays a vital role in virulence, probably at the earliest stages of bacterium-plant interaction. *glf* encoding UDP-galactopyranose mutase was disrupted in mutant M392. Glf is involved in the synthesis of galactofuranose, a component of the bacterial cell wall of human and animal pathogenic bacteria [50]. The role of Glf has not been assessed in plant-pathogenic bacteria yet, therefore we are unable to speculate about the possible function of *glf* gene product in *P. parmentieri*. Mutant M465 has a transposon insertion in *arnB* (*pmrH*) gene. The knowledge of the function of ArnB in the ecology and pathogenicity of SRP bacteria is limited. However, in *D. dadantii* strain 3937, the *arnB* gene encodes an enzyme modifying the lipid A structure with arabinose derivatives [51], providing protection of the bacterium during infection of aphids [52]. Similarly, the structural modifications of LPSs caused by the ArnB in other human and animal bacterial pathogens are speculated to provide protection from cationic antimicrobial peptides synthesized as a response to infection [53]. As plants are also known to produce cationic antimicrobial peptides as part of their innate immune system [54], it is reasonable to assume that the role of the ArnB in *P. parmentieri* may be linked with survival and protection of the bacterium during colonization of the host. The *wbjB* (*pseB*, *elgL*) gene, disrupted in mutant M603, encodes UDP-N-acetylglucosamine 4,6-dehydratase that is involved in the synthesis of both flagella and LPS in *H. pylori* and *C. jejuni* [55]. In these bacteria, a *wbjB*-deficient mutant lacked flagella and was nonmotile. However, in our study, mutant M603 possessed flagella and remained motile but apparently could not invade growing potato plants and cause disease symptoms. The last gene, mutated in the mutant M649 encodes hypothetical protein. The function of this protein could not be assessed due to the lack of homology with known protein sequences deposited in the international databases. In the genome of *P. parmentieri* SCC 3193 the gene disrupted by the transposon presence in the mutant M649 is present however in the same operon as the gene *glf*, mutated in the mutant M392. It is worth noticing that mutants M392 and M649 shared some phenotypes as demonstrated in our study.

Of the 7 bacterial transcriptional units analyzed in this study, 2 were predicted to be transcribed as individual genes (insertions: *p4* and *p26*) whereas 5 others (insertions: *p205*, *p392*, *p465*, *p603* and *p649*) were predicted to be transcribed as operons. It cannot be ignored therefore that the observed phenotypes of the mutants M205, M392, M465, M603 and M649 resulted not from inactivation of an individual gene (as in the case of *p4* and *p26* insertions) but rather from inactivation of the operon inside which the gene was located. In closely related bacteria (e.g., *E. coli* and *Klebsiella pneumoniae*), the Tn5 transposons were reported to influence the transcription of the genes located downstream to the insertion site in the operon [56,57]. Although polarity effects of Tn5 transposition in SRP bacteria has not been widely described to date [58] and no knowledge exists about such impact of transposon insertions in *P. parmentieri*, it should be presumed that the introduction of the Tn5 transposon into SCC 3193 chromosome will influence the transcription not only of the directly disrupted gene, but also may alter the transcription of the genes localized in the downstream of the insertion in the same operon. The knowledge of the molecular basis of *P. parmentieri* interactions with bacteriophages is scarce. As the genes in operons are in the majority functionally related to each other and regulated coordinately [59] it is interesting to see that in *P. parmentieri* strain SCC 3193 at least five of the operons involved in the synthesis of cell surface polysaccharides, as demonstrated in this study, are also involved in the interaction of SCC 3193 with its viral predator, bacteriophage ϕA38 (vB_Ppp_A38).

The inhibition of phage adsorption via modification of cell surface polysaccharides (e.g., LPS) has been frequently reported as one of the most common resistance mechanisms used to prevent phage infections in Gram-negative bacteria [60,61]. To date, however, only limited information is available on how often this mechanism is employed by SRP as a phage evasion strategy. We found that our phage-resistant mutants showed a substantial reduction (mutant M4) or complete inhibition (M26, M205, M392, M465, M603, and M649) in adsorption of ϕA38. This observation directly links the adsorption of ϕA38 with the cell surface polysaccharide features of *P. parmentieri*. The pivotal role of cell surface polysaccharides (e.g., LPS, capsule) in phage-host interactions has been described for several *Pectobacterium* spp. species including *P. atrosepticum* strain SCRI 1043 [62], *P. brasiliense* [63], and *P. carotovorum* strain Pcc27 [41] but so far not for the interaction of *P. parmentieri* and its bacteriophages. 

To further explore the possibility that LPS is the sole receptor needed by ϕA38 to infect *P. parmentieri* strain SCC 3193, we compared the LPS from phage-resistant mutants with that of the WT strain. All phage-resistant mutants except M26 produced statistically lower quantities of LPS than the SCC 3193 wild-type strain. Surprisingly, however, no direct correlation was found between the quantity of LPS isolated from phage-resistant mutants and the efficiency and/or velocity of attachment of ϕA38 to them. Likewise, the characterization of the LPSs by SDS-PAGE did not reveal, except for mutant M26, noticeable structural differences between that of the WT and mutant strains that could explain the ϕA38-resistant phenotype. These observations may suggest that it is likely that while LPS in *P. parmentieri* SCC 3193 mediates initial binding of ϕA38 particles to host cells, it is not the sole receptor required by this phage to infect SCC 3193. This hypothesis may be further strengthened by the fact that purified LPS could not inactivate ϕA38 particles in vitro.

It has been previously reported in some phage-host systems that LPS may serve only as a primary receptor that mediates the initial attachment of phage particles to host cells. Still, that phage must also recognize a secondary receptor (e.g., transport channel proteins or pili) to complete the transfer of its genetic material to the host’s cytoplasm [49,50,51]. Such a situation has been already reported for many phages, which bind to specific motives of the host LPS. These bacterial viruses require a secondary receptor to irreversibly bind to a host cell [64]. It cannot be excluded that the similar situation takes place in the case of attachment of ϕA38 to SCC 3193 cells.

Although phage-resistant mutants differed in the abundance and structure of the surface polysaccharides, mass spectrometry analyses of the cell surface did not show any significant alternations of the *P. parmentieri* cell surface proteins. The recorded MALDI-TOF spectra of the seven Tn5 mutants compared with the spectra recorded for the SCC 3193 wild-type strain revealed only minor differences in quantities but not in the qualities of the proteins present on the cell surface.

All phage-resistant *P. parmentieri* mutants were impaired in their ability to cause disease symptoms in planta. Although only 4 mutants had a reduced ability to macerate potato slices when directly inoculated on potato tuber slices in vitro, all mutants expressed reduced ability to colonize and cause symptoms in potato plants when presented to plants in soil under conditions typical of agricultural settings. Similar observations of LPS mutants have been made for other plant pathogens. For example, the LPS-defective mutants of *Ralstonia solanacearum* expressed reduced virulence in tobacco plants [65]. In *Dickeya* spp. (former *Erwinia chrysanthemi*), loss of virulence and unsuccessful plant colonization were reported for mutants with altered LPS structure [48]. Similarly, mutations in LPS caused reduced disease symptoms caused by *Erwinia amylovora* in pear [66].

The phage-resistant *P. parmentieri* mutants, while having altered polysaccharide synthesis, also expressed various phenotypes that are likely not directly related to phage-resistance but apparently contribute to their environmental fitness. Other studies of mutants with defects in LPS have revealed their role in resistance to different stresses [67,68]. Likewise, mutations resulting in alternations of the LPS structure were found to be associated with the altered ability to form biofilm and/or to survive under various abiotic stresses [68]. The most apparent phenotype of all mutants, except mutant M4, was impaired growth in an acidic environment but not at neutral and basic pHs. Other phenotypes observed, including altered swimming motility of mutants M26, M205, M392, and M465, as well as elevated biofilm formation detected for mutant M26, could be attributed to alterations in LPS biosynthesis. It is thus clear that LPS plays a central role both in the interaction of bacteria with their external world and viral predators and that modification of their LPS to avoid predators (phage-resistance) will likely impact the ecological fitness of their hosts. 

In conclusion, this study is, to our knowledge, the first to investigate the connection between phage-resistance and the ecological fitness of the plant-pathogenic *P. parmentieri* strain SCC 3193 in its plant environment (*S. tuberosum* plants). Although phage-resistance did not affect most of the phenotypes of the mutants screened in vitro, all seven SCC 3193 transposon mutants resistant to infection caused by vB_Ppp_A38 (ϕA38) were critically affected in their ability to cause disease symptoms in potato plants. They were either unable to infect plants after soil infestation with the bacteria or were able to internally colonize roots and move upward in the vascular tissue inside the stems, causing the systemic infection but without the manifestation of disease symptoms. Future work exploring phage-resistance in plant-pathogenic bacteria–plant hosts’ interactions is planned to assess in detail the adaptation and evolution of plant pathogens in phage-full natural environments.

## 4. Materials and Methods

### 4.1. Bacteriophages, Bacterial Strains and Growth Media

The lytic bacteriophage vB_Ppp_A38 (ϕA38) was isolated and characterized in detail previously [24,25]. For this work, ɸA38 was propagated on its wild-type bacterial host, *P. parmentieri* SCC 3193 [57], and titered as described earlier [15]. The adjusted stock concentration of ϕA38 phage particles was 10^8^–10^9^ plaque-forming units (PFU) mL^−1^ in tryptone soya broth (TSB, Oxoid, Basingstoke, UK) or quarter-strength (1/4) Ringer’s buffer (Merck, Warsaw, Poland) unless stated otherwise. *P. parmentieri* strain SCC 3193 (wild-type: WT) was grown for 24–48 h at 28 °C on tryptic soya agar (TSA, Oxoid, Basingstoke, UK), in tryptone soya broth (TSB, Oxoid, Basingstoke, UK) or in M9 minimal medium (MP Biomedicals, Santa Ana, CA, USA) supplemented with glucose (Sigma-Aldrich, Darmstadt, Germany) to a final concentration of 0.4%.When required, 15 g L^−1^ bacteriological agar (Oxoid, Basingstoke, UK ) was added to solidify the media and if needed the growth media were supplemented with neomycin (Sigma-Aldrich, Darmstadt, Germany) to a final concentration of 50 µg mL^−1^. The liquid bacterial cultures were agitated at 120 rpm during incubation. The phage-resistant *P. parmentieri* Tn5 mutants characterized in this study are listed in Table 1.

### 4.2. Transposon Mutagenesis with Mini-Tn5

Random transposon mutagenesis of *P. parmentieri* strain SCC 3193 was done as previously described [69,70] using *Escherichia coli* strain S17 λ-*pir* harboring pFAJ1819-miniTn5 plasmid, obtained from Belgian Coordinated Collections of Microorganisms-BCCM, Brussels, Belgium [71], as a transposon donor. The rate of Tn5 transposition (conjugal transfer from plasmid pFAJ1819mini-Tn5 in *E. coli* to *P. parmentieri*) was determined as described earlier [69]. To assess the coverage of the SCC 3193 genome with the Tn5 mutagenesis events in the assays, the Clark-Carbon equation [P = 1 − (1 − f)^N^] was applied [72], where P–is a probability to find a gene with the desired function, f–a fraction of the genome [if an average gene in *P. parmentieri* SCC 3193 is 1100 bp.-long and that the bacterial genome is 5,164,411 bp [36], then f = 1100/5,164,411 = 0.000213] and N–the number of constructed SCC 3193 Tn5 mutants (in our experimental design, N = 10,000) was used to determine the coverage of the SCC 3193 genome with transposition events as previously described [69].

### 4.3. Verification of Tn5 Mutants by Plating on CVP Medium and Selection of ΦA38-Resistant P. parmentieri Mutants

Phage-resistant *P. parmentieri* Tn5 mutants were preselected as previously described [73]. To validate their bacteriophage resistance, each Tn5 mutant exhibiting a ϕA38-resistant phenotype in the initial screen was subjected to repeatable phage challenge assays and plaque formation assays as previously described [15]. The ability of *P. parmentieri* Tn5 mutants to form cavities (pits) on a crystal violet pectate medium (CVP) was tested as previously described [74].

### 4.4. Kinetics of ΦA38 Adsorption to Viable P. parmentieri SCC 3193 and P. parmentieri Tn5 Mutants

The rate of ϕA38 adsorption to wild-type *P. parmentieri* SCC 3193 and selected Tn5 mutants was determined as previously described [15]. Briefly, log-phase grown wild-type *P. parmentieri* SCC 3193 or Tn5 mutant cells were infected with a phage suspension (MOI = 0.01) and incubated at 28 °C for up to 20 min. After 0 (control), 1, 2, 5, 10, 15, and 20 min, two individual samples per mutant were collected and centrifuged (10,000 *g* for 5 min) to sediment the bacteria as well as any attached phage particles. The subsequent supernatants were filter-sterilized with a 0.22 µm syringe filter (VWR International, Gdansk, Poland) to remove bacterial cells and assayed for free, unabsorbed phages. Negative control was bacteriophages suspended in sterile TSB incubated for 20 min under the same conditions as described above. The experiment was separately repeated three times with the same setup and the results were averaged. Phage adsorption was calculated using the formula: percentage adsorption = (the average titer of unabsorbed phages per sample/average titer of phages in negative control) × 100.

### 4.5. Kinetics of ΦA38 Adsorption to Chloramphenicol-Killed P. parmentieri SCC 3193

To test whether ϕA38 can adsorb to non-viable (dead) *P. parmentieri* SCC 3193 cells, the dead cell adsorption assay was employed [75]. Briefly, SCC 3193 was grown in TSB for 16 h at 28 °C with shaking (150 rpm). After overnight incubation, chloramphenicol (Sigma-Aldrich, Darmstadt, Germany) was added to the final concentration of 5 mg mL^−1^ to kill SCC 3193 cells. Such *P. parmentieri* cultures were incubated for another 1 h under the same conditions. To test the killing efficiency, 100 µL aliquots, in duplicate, were collected, plated on a TSA agar plate, and incubated at 28 °C to allow colonies to grow. The chloramphenicol-killed *P. parmentieri* SCC 3193 cells were infected with a phage suspension (at MOI = 0.01) and assayed for phage adsorption as described above. The experiment was repeated three times using the same procedure, and the results were averaged. Phage adsorption was calculated similarly as described above using the formula: percentage adsorption = (the average titer of unabsorbed phages per sample/average titer of phages in negative control) × 100.

### 4.6. Identification of the Tn5 Insertion Sites in Phage-Resistant Mutants by Genome Sequencing

To preciously localize the Tn5 insertion sites in the genomes of *P. parmentieri* mutants, the genomes of selected mutants were sequenced and analyzed. The genomic DNA of each bacteriophage-resistant mutant was isolated, sequenced, and assembled into a draft genome at the Laboratory of DNA Sequencing and Oligonucleotide Synthesis (Institute of Biochemistry and Biophysics of the Polish Academy of Science, Warsaw, Poland) using Illumina technology. Both structural and functional annotations of draft Tn5 genomes were acquired from RAST (Rapid Annotation using Subsystem Technology (http://rast.nmpdr.org/ accessed on 3 December 2020) [76]. The position of the Tn5 insertions in the draft genomes of *P. parmentieri* SCC 3193 Tn5 mutants was determined using BlastN and BlastX alignments (http://blast.ncbi.nlm.nih.gov/Blast.cgi accessed on 4 November 2020) [68].

Using the available complete genome sequence of *P. parmentieri* strain SCC 3193 (Genbank accession: CP003415.1) [36] and the draft genomes of the Tn5 mutants, the insertion of the Tn5 transposon in the bacterial chromosome was assessed in detail as described earlier [69]. For each mutant, at least ca. 1000- to 5000-bp-long sequences bordering the Tn5 insertion site were analyzed to determine the genomic context of each of the Tn5-disrupted genes [69,77]. The presumed function of the disrupted genes was inferred using BlastN and BlastX alignments accessed as described above. Likewise, the functions of any unannotated open reading frames encoding hypothetical proteins or proteins without homology to known proteins were analyzed using GeneSilico Protein Structure Prediction meta-server, containing protein structures [37,78], together with PSI-BLAST [71] accessed from the NCBI website. The predicted functions with the highest scores obtained were judged as the most probable.

### 4.7. The Transcriptional Organization, Biochemical Pathways and Cellular Enzymatic Networks Affected by the Transposon Insertions into P. parmentieri Genomes

The putative transcriptional organization of *P. parmentieri* SCC 3193 genes disrupted by Tn5 was determined using Operon-mapper (https://biocomputo.ibt.unam.mx/operon_mapper/ accessed on 26 April 2021) [79]. The complete genome sequence of *P. parmentieri* SCC 3193 was used as a reference. Inference of the biochemical pathways in which the genes of interest might participate was made using KEGG [80]. The results were visualized using iPath [81]. Likewise, proteins were evaluated for their predicted biological, functional, and metabolic roles in cellular networks using STRING (Search Tool for Retrieval of Interacting Genes/Proteins) v11 accessed via the website (https://string-db.org/ accessed on 27 April 2021), providing essential information regarding interactions of proteins of interest [82] using the proteome of *P. atrosepticum* strain SCRI1043 (the closest possible match for *P. parmentieri* strain SCC 3193) [83] as a reference.

### 4.8. Extraction, Quantification, and Visualization of Lipopolysaccharide (LPS) from Wild-Type P. parmentieri Strain SCC 3193 and Selected Phage-Resistant P. parmentieri Tn5 Mutants

Lipopolysaccharide (LPS) of *P. parmentieri* SCC 3193 wild-type strain and the LPS of selected phage-resistant Tn5 mutants was isolated with a LPS Extraction Kit (Abcam, Symbios, Gdansk, Poland) using a modified protocol. In brief, the bacterial cultures grown as described above were collected from TSA plates using sterile cotton swabs and individually resuspended in 12 mL of phosphate buffer saline (PBS, pH 7.2, Sigma-Aldrich, Darmstadt, Germany). Bacterial suspensions were centrifuged (2500× *g*) for 5 min at 4 °C, and the supernatant was removed. The resulting bacterial pellets were washed two times with equal volumes of PBS and under the same conditions. After the second wash, the pellets were individually suspended in 100 µL of quarter-strength Ringer’s buffer, placed on ice, and weighted. Per sample, a volume of LPS lysis buffer equal to 20 × the weight of the individual bacterial pellet was added to each pellet following its careful resuspension in the lysis buffer. The samples were incubated on ice for 10 min, centrifuged (10,000× *g*) for 10 min at 4 °C, and the subsequent supernatants were collected. The samples were incubated for 1 h at 60 °C in the presence of proteinase K (final concentration 0.5 mg mL^−1^ per sample) (Sigma-Aldrich, Darmstadt, Germany) to digest bacterial proteins. After incubation, the LPS samples were stored at 4 °C until further use. The experiment was repeated three time (three biological replicates per strain) and the results were averaged. The LPS concentration was quantified using a Pierce™ LAL Chromogenic Endotoxin Quantitation Kit (ThermoFisher Scientific, Warsaw, Poland) according to a protocol provided by the manufacturer. The quantity of LPS was normalized each time to 1 mg of fresh bacterial weight. LPS was separated using gradient 4–20% sodium dodecyl sulfate-polyacrylamide gel (4–20% Mini-PROTEAN^®^ TGX™ Precast Protein Gel, BioRad, Hercules, CA, USA) electrophoresis (SDS-PAGE) according to standard methods [84] and visualized with silver staining as described elsewhere [85].

### 4.9. Inactivation of ΦA38 by Lipopolysaccharide (LPS) Isolated from Wild-Type P. parmentieri SCC 3193 Cells

The ability of the lipopolysaccharide isolated from a wild-type *P. parmentieri* SCC 3193 to inactivate ϕA38 phage particles was tested as previously described [86] with minor modifications. Briefly, 300 µL of purified undiluted LPS or LPS diluted 2, 4, 8, 16, and 32-fold in Ringer’s buffer was mixed with 100 µL of ɸA38 suspension (10^5^ PFU mL^−1^) and incubated for 1 h at 22 °C with gentle shaking (50 rpm). The mixtures were then serially diluted with Ringer’s buffer and assayed for bacteriophage presence using a soft top agar assay with *P. parmentieri* SCC 3193 as a host as previously described [15].

### 4.10. Determination of the Generation Time of P. parmentieri Tn5 Mutants in Rich and Minimal Media

To determine whether the Tn5 insertions affected the generation time of the mutants, the growth of the selected *P. parmentieri* Tn5 mutants was assessed in TSB (rich medium) as well as in M9 supplemented with 0.4% glucose (minimal medium) at 28 °C for 16 h as previously described [70]. Briefly, overnight bacterial cultures with a density of ca. 10^9^ colony-forming units (CFU) mL^−1^ in TSB or M9 + 0.4% glucose were diluted 50-fold in a fresh medium. One hundred microliter aliquots of diluted bacterial culture were aseptically transferred to the wells of 96-well microtiter plates (NEST, Wuxi, China) and sealed with optically clear sealing tape (Sarstedt, Warsaw, Poland) to prevent desiccation of bacterial culture. The growth rate of the Tn5 mutants was determined by measuring the optical density (OD) (λ = 600 nm) every 30 min in an Epoch2 microplate spectrophotometer (BioTek, Colmar, France) for a total time of 16 h as previously described [69]. The growth of each mutant was analyzed in three technical replicates, and the results were averaged. Each 96-well plate contained three negative (sterile growth medium) and three positive (wild-type *P. parmentieri* SCC 3193 culture) wells as controls. The experiment was separately repeated once, and the results were averaged. The average generation time was calculated using the Doubling Time calculator (parameters: C0 = 3 h, Ct = 7 h, t = 4 h) (http://www.doubling-time.com/compute.php accessed on 28 April 2021) [87].

### 4.11. Determination of the Average Generation Time of P. parmentieri Tn5 Mutants at pH 5 and pH 10

To test whether the Tn5-mediated phage-resistance affected the fitness of the mutants grown at low and/or high pHs, the growth of selected *P. parmentieri* Tn5 mutants was assessed in TSB at pH 5 and TSB at pH 10, similar to other studies [88]. Briefly, overnight bacterial cultures with a density of ca. 10^9^ CFU mL^−1^ in TSB were diluted 50-fold in a fresh medium (TSB pH 5 or TSB pH 10). One hundred microliters of diluted bacterial culture were aseptically transferred to the wells of 96-well microtiter plates (NEST, Wuxi, Jiangsu, China) and sealed with optically clear sealing tape (Sarstedt, Warsaw, Poland) to prevent evaporation. The bacterial growth was determined by optical density (OD) (λ = 600 nm) measured every 30 min for 12 h in an Epoch2 microplate spectrophotometer (BioTek). The experiment was reproduced with two biological replicates each containing two technical replicates (*n* = 4), and the obtained results averaged. The generation time was calculated using the Doubling Time calculator (parameters: C0 = 3 h, Ct = 7 h, t = 4 h) (http://www.doubling-time.com/compute.php accessed on 29 April 2021) [87]. 

### 4.12. Growth of Phage-Resistant Tn5 Mutants at Different Temperatures on Solid Media

The growth of phage-resistant Tn5 mutants and the wild-type strain was tested on solid media (TSA and M9 + 0.4% glucose) over a range of six temperatures: 5, 8, 15, 28, 37, and 42 °C as described earlier [89]. A total of 5-µL aliquots of 50-fold diluted in TSB or M9 + 0.4% glucose overnight bacterial cultures grown in either TSA and M9 + 0.4% glucose, respectively, were placed on the surface of either TSA or M9 + 0.4% glucose and incubated for 120 h at 5 and 8 °C or for 48 h for all other temperatures. Growth was assessed visually. The experiment was repeated once, and the results averaged.

### 4.13. Antibiotic Susceptibility of Tn5 Mutants

The antibiotic susceptibility of *P. parmentieri* Tn5 mutants was determined by a disc diffusion method, as previously described [90]. Antibiotic discs (BD BBL-Sensi-Disc antimicrobial test discs) used in this study were: chloramphenicol (30 µg), gentamicin (10 µg), tigecycline (15 µg), doxycycline (30 µg), sulfametoxazol/trimetropin (23,75/1.25 µg), ciprofloxacin (5 µg), ceftaroline (5 µg), imipenem (10 µg), piperacillin/tazobactam (30/6 µg), cefuroksym/ceftaroline (30/5 µg), cefuroxime (30 µg), aztreonam (30 µg), ampicillin (10 µg), ampicillin/sulbactam (10/10 µg), colistin (10 µg), fosfomycin (200 µg). Briefly, the wild-type *P. parmentieri* and phage-resistant *P. parmentieri* Tn5 mutants were grown for 16 h in TSB (WT strain) or TSB supplemented with neomycin (50 μg mL^−1^) as appropriate, at 28 °C with shaking (120 rpm). The Mueller-Hinton (MH medium, BD, Warsaw, Poland) supplemented with 1.5% agar (Oxoid, Basingstoke, UK) square plates (100 × 100 mm^2^) were inoculated using a sterile cotton swab (Sarstedt, Warsaw, Poland) soaked in a suspension of individual strains. Once the inoculated MH plates had dried, the antibiotic discs were placed on the agar surface in a way to ensure a minimum distance between each disc of ca. 2 cm. Plates were then incubated for 24 h at 28 °C and subsequently screened for the presence of a clear halo in the bacterial lawn around the discs. The presence of the halo was recorded as a negative reaction (bacterial susceptibility), whereas the lack of halo was registered as a positive reaction (bacterial resistance). Wild-type *P. parmentieri* strain SCC 3193 was used as a control. The experiment was repeated once.

### 4.14. Phenotypic Characterization of P. parmentieri Tn5 Mutants Using BIOLOG Phenotypic Microarrays

Selected phage-resistant *P. parmentieri* SCC 3193 mutants were analyzed with the BIOLOG phenotypic microarray system with GEN III and EcoPlate microplates (Biolog Inc.). Each GEN III plate contained 94 phenotypic tests, i.e., 71 carbon-source use assays and 23 chemical sensitivity assays. Each EcoPlate contains 31 different complex carbon sources (BIOLOG, Hayward, CA, USA). Before analysis, bacterial cultures were grown on TSA plates for 24 h at 28 °C, and then resuspended into inoculation fluid (IF-A) (GENIII) or into 10 mM phosphate buffer pH (EcoPlate) using a sterile cotton swab. The turbidity of the bacterial cell suspension was adjusted to ca. 90% T with a spectrophotometer [A = log(%T)] as suggested by the manufacturer. One hundred microliters of suspensions in duplicates were inoculated into each well of the microplates using a multichannel pipette. Inoculated plates were sealed with optically clear sealing tape (Sarstedt, Warsaw, Poland) and incubated for 24 h at 28 °C. The wells were then examined for a color change. Color development was also recorded using an Epoch2 microplate spectrophotometer (BioTek, Colmar, France) equipped with a λ = 595-nm wavelength filter. Plates inoculated with the wild-type *P. parmentieri* strain SCC 3193 WT were used as controls.

### 4.15. Phenotypic Characterization of Tn5 Mutants Using Plate Assays

ɸA38-resistant *P. parmentieri* Tn5 mutants were screened for various phenotypic features, putatively important for their interaction with plant tissues, including swimming and swarming motility [88], biofilm formation [91], the ability to grow on the TSA supplemented with 5% NaCl [92], ability to grow in TSB at different pHs [88], production of enzymes: cellulases [93], proteases [93], pectinolytic enzymes [94] and siderophores [95]. The phenotypic tests were done at 28 °C. 

### 4.16. Assessment of Bacterial Cell and Colony Morphology of P. parmentieri Mutants

The morphology of bacterial cells was evaluated using transmission electron microscopy (TEM), as previously described [70]. TEM analysis was performed at the Laboratory of Electron Microscopy (Faculty of Biology, University of Gdansk, Gdansk, Poland). Bacteria were adsorbed onto carbon-coated grids (GF Microsystems, Poznan, Poland), stained with 1.5% uranyl acetate (Sigma-Aldrich), and directly analyzed with an electron microscope (Tecnai Spirit BioTWIN, FEI, New York, NY, USA) as described previously [70]. At least ten images were taken per each mutant and the wild-type strain to estimate cell diameter. Similarly, the morphology of bacterial colonies of selected phage-resistant *P. parmentieri* SCC 3193 Tn5 mutants was analyzed using a Leica MZ10F stereomicroscope with 10× and 40× magnifications combined with a Leica DFC450C camera (Leica, Wetzlar, Germany) as previously described [69].

### 4.17. Interaction of ΦA38 with SCC 3193 and SCC 3193 Tn5 Mutants Assessed with Transmission Electron Microscopy (TEM)

To visually assess the ability of ϕA38 to attach to the host bacterial surface, transmission electron microscopy (TEM) was used. Different volumes of ϕA38 suspension (ca. 10^9^ PFU mL^−1^) in TSB and fresh bacterial culture (ca. 10^8^ CFU mL^−1^) washed three times and suspended in filter-sterilized 1/4 Ringer’s buffer were mixed and incubated at 20–22 °C for 20 min to allow the phages to find and attach to bacterial cell surfaces before TEM analyses. The bacteria and phage particles were then adsorbed onto carbon-coated grids (CF300-CU, Electron Microscopy Sciences, Hatfield, PA, USA), stained with 1.5% uranyl acetate (Sigma-Aldrich, Darmstadt, Germany), and examined with an electron microscope (Tecnai Spirit BioTWIN, FEI, New York, NY, USA) as described above. At least ten images were taken per each mutant and the wild-type strain.

### 4.18. Visualization of Capsule and Flagella of P. parmentieri Tn5 Mutants with Microscopic Techniques

To visualize bacterial capsules, SCC 3193 wild-type and Tn5 mutant cells were stained using Maneval’s method [96]. The presence of flagella was visualized as described earlier [97]. Stained bacterial cells were observed using light microscopy with standard settings [98].

### 4.19. Phenotypic Characterization of P. parmentieri Tn5 Mutants Using Whole-Cell MALDI-TOF MS Analyses

*P. parmentieri* SCC 3193 Tn5 phage-resistant mutants were tested using a whole-cell MALDI-TOF MS spectral analysis as previously described [59,93]. Briefly, SCC 3193 wild-type and Tn5 mutants were grown on M9 medium + 0.4% glucose at 28 °C for 24 h. Viable cells were loaded with a sterile loop directly from the culture onto a MALDI plate. Sinapinic acid (SA) (10 mg/mL) in 50% acetonitrile, 50% water and 0.1% trifluoroacetic acid (TFA) was used as a matrix. 1 µL of matrix solution was used to overlay the sample spot, and the plate was then left to crystallize at room temperature. After preparation of the spots (ca. 15 min), protein mass fingerprints were obtained using a 5800 MALDI-TOF/TOF mass spectrometer (AB Sciex, Framingham, MA, USA), with detection in the linear middle mass (4000–20,000 Da), positive ion mode for a total of 1000 laser shots with a 1 kHz OptiBeam laser (YAG, 349 nm). Laser intensity was corrected for all tested samples. Registered spectra were analyzed with Data Explorer software (AB Sciex). All MALDI-TOF MS spectra used in this study were averages of six replicated measurements (2 independent measurements each containing 3 technical repetitions) per analyzed strain.

### 4.20. The Ability of P. parmentieri Tn5 Mutants to Macerate Potato Tuber Slices

Potato tubers of cv. Bryza were obtained locally in Gdansk, Poland. Three individual potato slices obtained from three different potato tubers were inoculated with a given mutant and assessed for infection symptoms, as described before [88]. Wild-type *P. parmentieri* SCC 3193 was used as a positive control, and the negative control was sterile demineralized water. In the first experiment, two replicates of 3 potato slices derived from different tubers were assessed per mutant. In a second experiment, the mutants with rotting abilities statistically different from the wild-type SCC 3193 strain were repeatedly tested using the same protocol, as described above. The results from all repetitions were averaged.

### 4.21. Virulence of P. parmentieri Tn5 Mutants in Potato Plants Grown in a Growth Chamber

Duplicate experiments of plants grown in a growth chamber were performed as previously described [99]. The certified, pathogen-free potato tubers of cv. Kondor were obtained from Plant Breeding and Acclimatization Institute–National Research Institute, Bonin, Poland. Potato tubers were stored at 4 °C and 80% relative humidity in the dark until sprouting (ca. 1 ± 2 months). 5 ± 10 cm long sprouts were collected and placed in water-soaked peat pellets (Jiffy, Vilmorin, Komorniki, Poland) in a humid box in a growth chamber incubator at 22 °C under white fluorescent light with a 16 h photoperiod for rooting [99]. After two weeks, rooted plants with a height of ca. 10–15 cm were transferred to 2 L pots and grown in potting compost soil for another 2 weeks under similar conditions. Plants were watered 1 h before the infestation of soil with bacteria. Potato plants (*n* = 10 per treatment, 5 plants per repetition) were infected with bacterial strains by applying bacterial cultures (50 mL of 10^8^ CFU mL^−1^ bacterial suspension in sterile 1/4 Ringer’s buffer) to the soil. As a negative control, plants were watered with 50 mL of sterile Ringer’s buffer. The experiment was repeated once. Pots were randomized in the growth chamber: 5 blocks of 9 pots per treatment (*n* = 45 plants per repetition), and the experiment was repeated once (total *n* = 90 plants).

Plants were visually inspected daily for symptoms including wilting, chlorosis, stem black rotting, haulm desiccation, and plant death. Plants were sampled at 14 dpi (days post-inoculation) by excising ca. 2 cm long stem segments, located ca. 5 cm above ground level, and pooled per analyzed plant. The stem segments were surface-sterilized, as described before [99]. The presence of *P. parmentieri* cells was determined by plating the plant extracts on CVP (*P. parmentieri* SCC 3193 wild-type) and/or TSA supplemented with 50 μg mL^−1^ neomycin (Tn5 mutants) and counting the bacterial colonies. The neomycin-resistant colonies were additionally transferred to the CVP medium to verify that they produced cavities (pits) typical of SRP bacteria [74].

### 4.22. Statistical Analyses

Bacterial colony numbers were transformed as log(x + 1). The Shapiro–Wilk test (*p* < 0.05) [100] was used to test the normality of results distribution for individual counts, whereas for populations of counts not equally distributed between the analyzed groups (control vs. treatment and/or treatment vs. treatment) the Welch’s T-test [101] was applied. The homogeneity of variance was validated by the Fisher–Snedecor test [102]. Pair-wise differences were assessed using a two-tailed Student’s *t*-test [103]. The in vitro grown plants were analyzed according to the experimental design in which two replicated experiments (*n* = 5 per treatment and per experiment, *n* = 10 per treatment) were done per each treatment of replicated plants. The implemented linear model was a complete block design with replicates as blocks. The main effects were analyzed for the contribution of time and treatment and a two-way interaction between time and treatment. As described earlier, a normal distribution of plant height and weight was assumed [77,104].

## Figures and Tables

**Table 1:** Genetic loci of *Pectobacterium parmentieri* strain SCC 3193 Tn5 mutants expressing resistance against ϕA38 phage.

No	Mutant ^a^	Insertion Name, Tn5 Locus, Gene, CDC	Protein Name	Transcriptional Organization (Single Gene vs. Operon) ^b^	Entry, KEGG Pathway, UniProt-Based Protein Function
1	**M4**	***p4***, putative *oafA*, W5S_2104, AFI90193.1	O-acetyl transferase (putative O-antigen LPS acetylase)	single gene	EC: entry not assigned, gene not included in the pathway
2	**M26**	***p26***, *galU*, W5S_2225, AFI90313.1	UTP-glucose-1-phosphate uridylyl transferase	single gene	EC: 2.7.7.9, O-antigen nucleotide sugar biosynthesis, amino sugar, and nucleotide sugar metabolism
3	**M205**	***p205***, *pseH*, W5S_3016, AFI91099.1	pseudaminic acid biosynthesis N-acetyl transferase	operon: contains 16 genes	EC: 23.1.202, O-antigen nucleotide sugar biosynthesis, amino sugar, and nucleotide sugar metabolism
4	**M392**	***p392***, *glf*, W5S_3004, AFI91087.1	UDP-galactopyranose mutase	operon: contains 2 genes	EC: 5.4.99.9, O-antigen nucleotide sugar biosynthesis, amino sugar and nucleotide sugar metabolism
5	**M465**	***p465***, *arnB*, W5S_3019, AFI91102.1	UDP-4-amino-4-deoxy-L-arabinose-oxoglutarate aminotransferase	operon: contains 8 genes	EC: 2.6.1.87, amino sugar and nucleotide sugar metabolism
6	**M603**	***p603***, *wbjB* (synonyms: *pseB*, *elgL*), W5S_3021, AFI91104.1	UDP-N-acetylglucosamine 4,6-dehydratase	operon: contains 16 genes	EC: 42.1.115, O-antigen nucleotide sugar biosynthesis
7	**M649**	***p649***, W5S_3005, AFI91088.1	hypothetical protein	operon: contains 2 genes	EC: 5.4.99.9, O-antigen nucleotide sugar biosynthesis, amino sugar and nucleotide sugar metabolism

^a^ Draft genome sequences of the seven Tn5 phage-resistant mutants (M4, M26, M205, M392, M465, M603 and M649) used to in detail localize the transposon insertions in the genome of *P. parmentieri* strain SCC 3193 are attached as Supplementary Material as FASTA files. ^b^ Assessment of the transcriptional organization was predicted using Operon-mapper (https://biocomputo.ibt.unam.mx/operon_mapper/ accessed on 9 June 2021). The complete genome sequence of *P. parmentieri* strain SCC 3193 (Genbank accession CP003415.1) [36] was used as a reference.

## Data Availability

Data are contained within the article and Supplementary Materials.

## Supplementary Materials

Supplementary materials can be found at https://www.mdpi.com/article/10.3390/ijms22147346/s1.
