# Supplementary material for: Pectobacterium parmentieri SCC 3193 Mutants with Altered Synthesis of Cell Surface Polysaccharides Are Resistant to N4-Like Lytic Bacteriophage ϕA38 (vB_Ppp_A38) but Express Decreased Virulence in Potato (Solanum tuberosum L.) Plants
Source: Int J Mol Sci. 2021 Jul 8;22(14):7346. doi: 10.3390/ijms22147346 (PMC8304393; doi:10.3390/ijms22147346)
Supplement: Supplementary file 1 [file ijms-22-07346-s001.zip › Supplementary Materials/Supplementary Materials.pdf]

Supplementary Materials:

Pectobacterium parmentieri SCC 3193 Mutants with Altered Synthesis of Cell Surface Polysaccharides are Resistant to N4-Like Lytic Bacteriophage ϕA38 (vB_Ppp_A38) but Express Decreased Virulence in Potato (*Solanum tuberosum* L.) Plants

Przemyslaw Bartnik [1], Sylwia Jafra [2], Magdalena Narajczyk [3], Paulina Czaplewska [4] and Robert Czajkowski [1,*]

[1] Laboratory of Biologically Active Compounds, Intercollegiate Faculty of Biotechnology UG and MUG, University of Gdansk, Antoniego Abrahama 58, 80-307 Gdansk, Poland; bartnikprzemyslaw@gumed.edu.pl

[2] Laboratory of Plant Microbiology, Intercollegiate Faculty of Biotechnology UG and MUG, University of Gdansk, Antoniego Abrahama 58, 80-307 Gdansk, Poland; sylwia.jafra@ug.edu.pl

[3] Laboratory of Electron Microscopy, Faculty of Biology, University of Gdansk, Wita Stwosza 59, 80-308, Gdansk, Poland; magdalena.narajczyk@ug.edu.pl

[4] Laboratory of Mass Spectrometry-Core Facility Laboratories, Intercollegiate Faculty of Biotechnology UG and MUG, University of Gdansk, Antoniego Abrahama 58, 80-307 Gdansk, Poland; paulina.czaplewska@ug.edu.pl

* Correspondence: robert.czajkowski@ug.edu.pl; Tel.: +0048-58-523-63-33

Supplementary Material. Draft genome sequences of the seven Tn5 phage-resistant mutants (M4, M26, M205, M392, M465, M603 and M649) used to in detail localize the Tn5 transposon insertions in the genome of *P. parmentieri* strain SCC3193. Draft genome sequences are in FASTA format and compressed with Windows Zip.

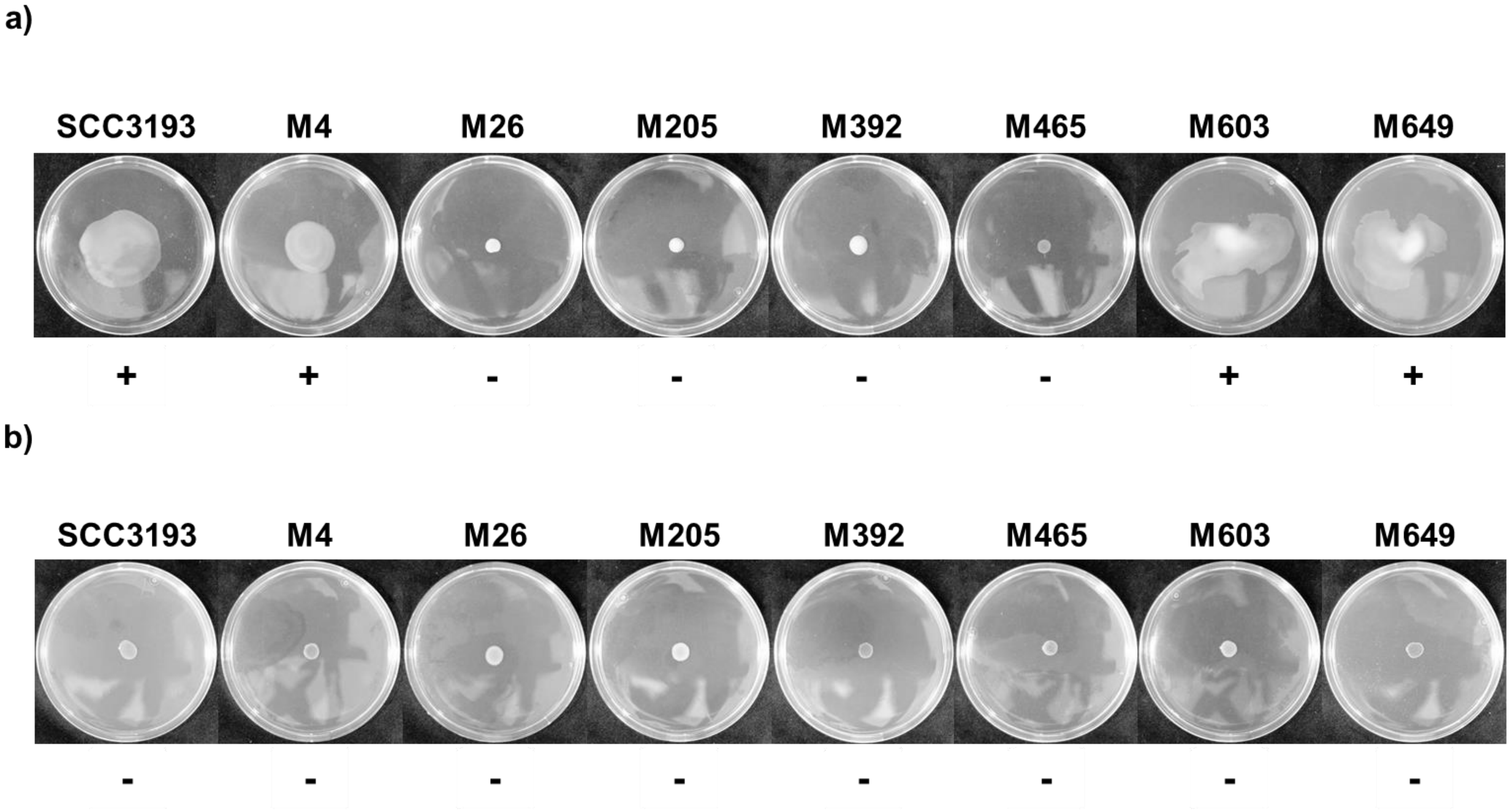

Figure S1. Assessment of swimming (a) and swarming (b) motility of SCC3913 WT strain and seven phage-resistant mutants (M4, M26, M205, M392, M465, M603 and M649. Representative photos are shown. (+) – indicates motility, (−) – indicates the lack of motility.

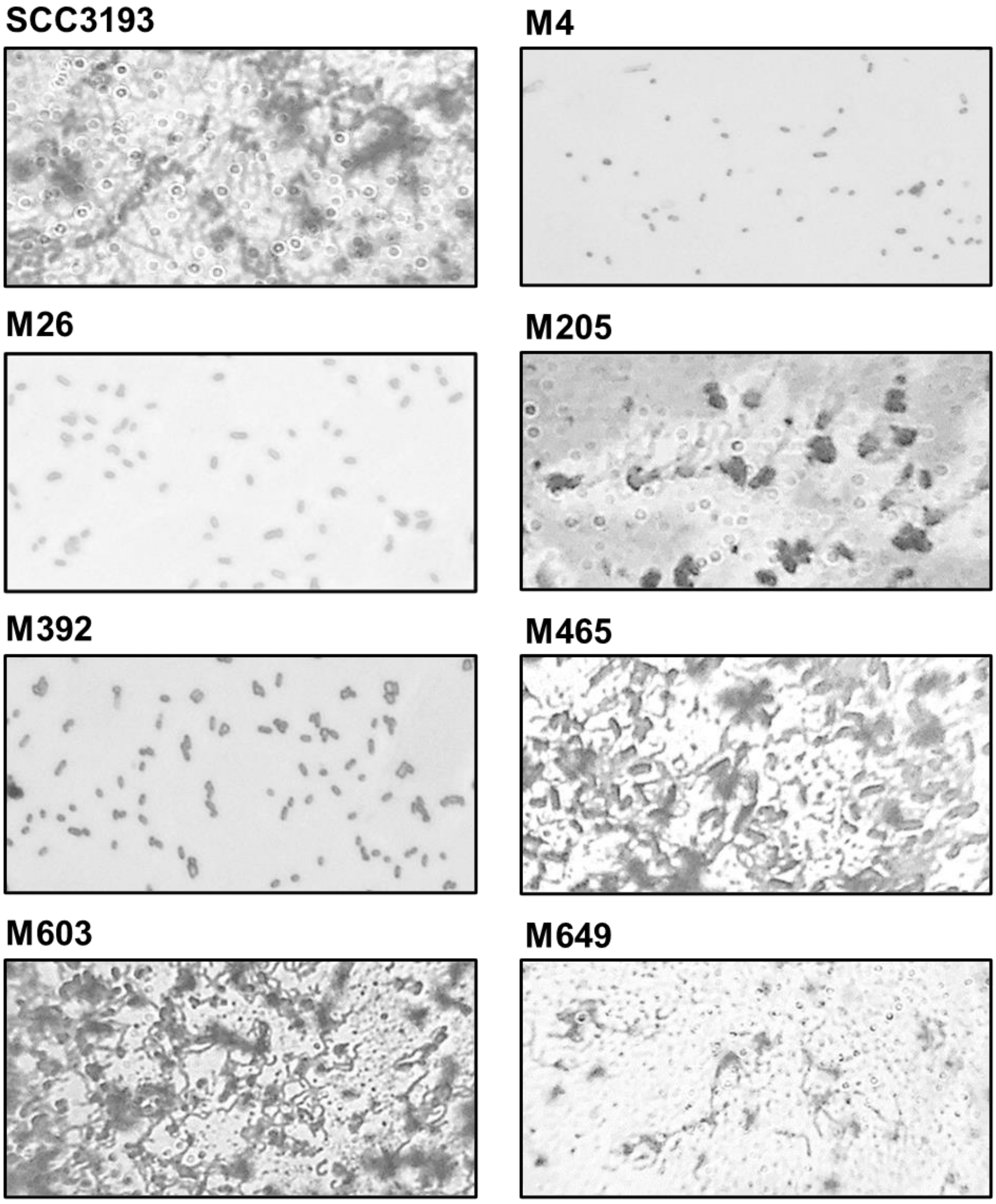

Figure S2. Visualization of the presence/absence of flagella of SCC3913 WT strain and seven phage-resistant mutants (M4, M26, M205, M392, M465, M603 and M649). Flagella staining was done using Ryu staining solution as described in [97]. Representative photos are shown.

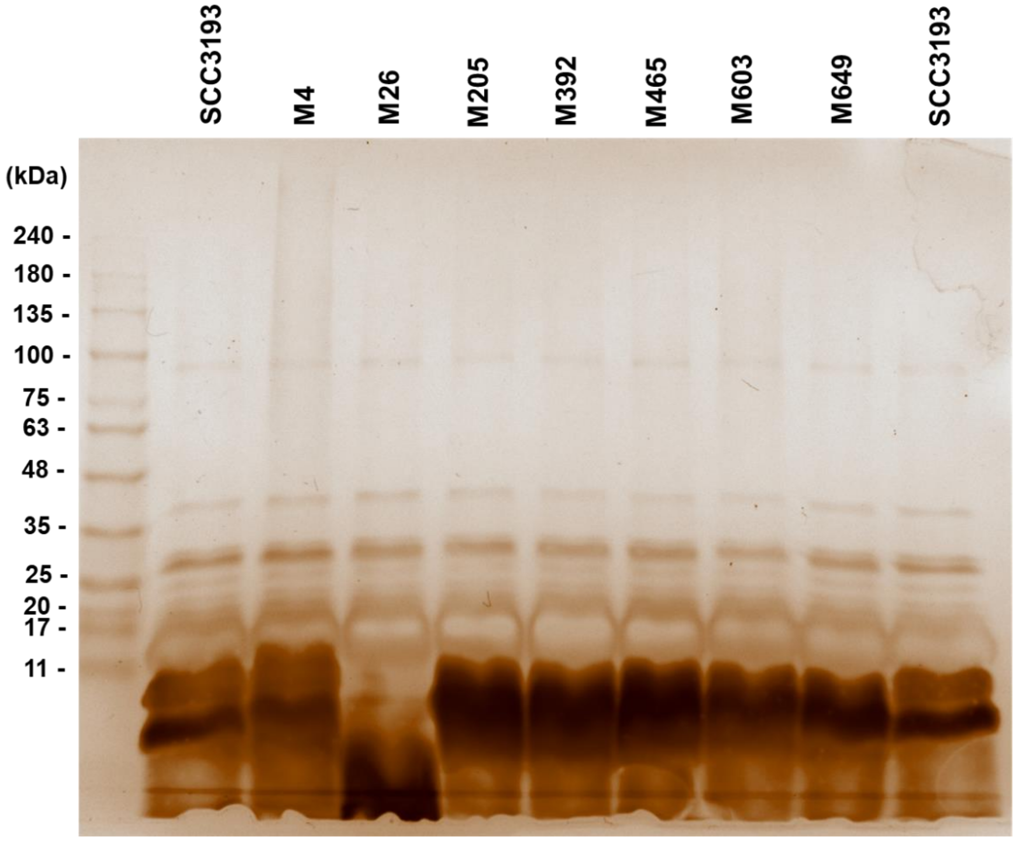

Figure S3. Characterization of lipopolysaccharide from wild type *P. parmentieri* strain SCC 3193 and seven phage-resistant mutants. (**a**) SDS-PAGE was performed using a gradient (4–20 %) polyacrylamide gel, and the LPS components were visualized by silver staining [77]. The size marker (11-245 kDa, Perfect Tricolor Protein Ladder, EURx, Poland) is shown in the first lane.
